# An in vivo evaluation of bone response to three implant surfaces using a rabbit intramedullary rod model

**DOI:** 10.1186/1749-799X-5-57

**Published:** 2010-08-16

**Authors:** Juan C Hermida, Arnie Bergula, Fred Dimaano, Monica Hawkins, Clifford W Colwell, Darryl D D'Lima

**Affiliations:** 1 Orthopaedic Research Laboratories, Shiley Center for Orthopaedic Research and Education at Scripps Clinic, 11025 North Torrey Pines Road, Suite 140, La Jolla, CA, 92037, USA; 2 Stryker Orthopaedics, 300 Commerce Court, Mahwah, NJ 07430, USA

## Abstract

Our study was designed to evaluate osseointegration among implants with three surface treatments: plasma-sprayed titanium (P), plasma-sprayed titanium with hydroxyapatite (PHA), and chemical-textured titanium with hydroxyapatite (CHA). Average surface roughness (Ra) was 27 microns for the P group, 17 microns for the PHA group, and 26 microns for the CHA group. Bilateral distal intramedullary implants were placed in the femora of thirty rabbits. Histomorphometry of scanning electron microscopy images was used to analyze the amount of bone around the implants at 6 and 12 weeks after implantation. Greater amounts of osseointegration were observed in the hydroxyapatite-coated groups than in the noncoated group. For all implant surfaces, osseointegration was greater at the diaphyseal level compared to the metaphyseal level. No significant differences were seen in osseointegration between the 6 and 12 week time points. Although the average surface roughness of the P and the CHA groups was similar, osseointegration of the CHA implants was significantly greater. The results of this in vivo lapine study suggest that the presence of an hydroxyapatite coating enhances osseointegration despite similarities in average surface roughness.

## Introduction

Total hip arthroplasty (THA) is a relatively common procedure that typically results in increased comfort, mobility, pain relief, and alleviation of disability. Once thought to be appropriate for patients between 60 and 75 years of age, the age range for primary THA now often includes a substantially younger population [1-4]. The procedure has an excellent clinical outcome and often restores functional capacity to a large degree. However, aseptic loosening of the components continues to limit the longevity of THA, especially in younger more active patients [1-11]. With the increase in life expectancy and the increase in younger patients undergoing primary THA, the need to extend the longevity of THA is essential.

Non-cemented THA offers the potential for integration of the implant surface with the surrounding bone. Hydroxyapatite coatings have proven effective in providing excellent short- and intermediate-term outcomes in terms of fixation, stability, function, and pain relief [12-17]. Hydroxyapatite coatings enhance osteoblast attachment, proliferation, and differentiation (see Beck for review [18]). While hydroxyapatite is generally considered to be an osteoconductive material, it has occasionally been shown to have osteoinductive properties, which have been attributed to the adsorption of bone morphogenetic proteins [19].

Osteoblastic activity is modulated by surface roughness and is enhanced when the R_a _is between 1 and 7 μm [20,21]. In addition, surface roughness in vivo is an important factor affecting bone apposition and mechanical strength of the implant-bone interface. Increasing surface roughness by grit-blasting or chemical-etching has been associated with increased osseointegration in a variety of animal models [22-25].

Since hydroxyapatite coating can alter surface roughness, it is important to determine the relative significance of the individual contributions of these factors [22,26]. For example, superior osseointegration was found in hydroxyapatite-coated trabecular implants in miniature pigs compared to grit-blasted or acid-etched surface [25]. However, the hydroxyapatite-coated implants had a significantly greater R_a_. It has not been conclusively shown whether surface roughness or hydroxyapatite coating is the dominant factor affecting in vivo osseointegration. One study concluded that surface roughness contributed more to increased bone apposition rates than hydroxyapatite coating [26]. On the other hand another study found significantly increased bone apposition in hydroxyapatite-coated implants despite comparable surface roughness measures between coated and uncoated implants [27]. We therefore designed a study to investigate the factors contributing to osseointegration in orthopedically relevant surfaces. The study hypothesis was that the addition of a hydroxyapatite coating would enhance osseointegration beyond that provided by change in surface roughness alone.

## Methods

**Figure 1:**
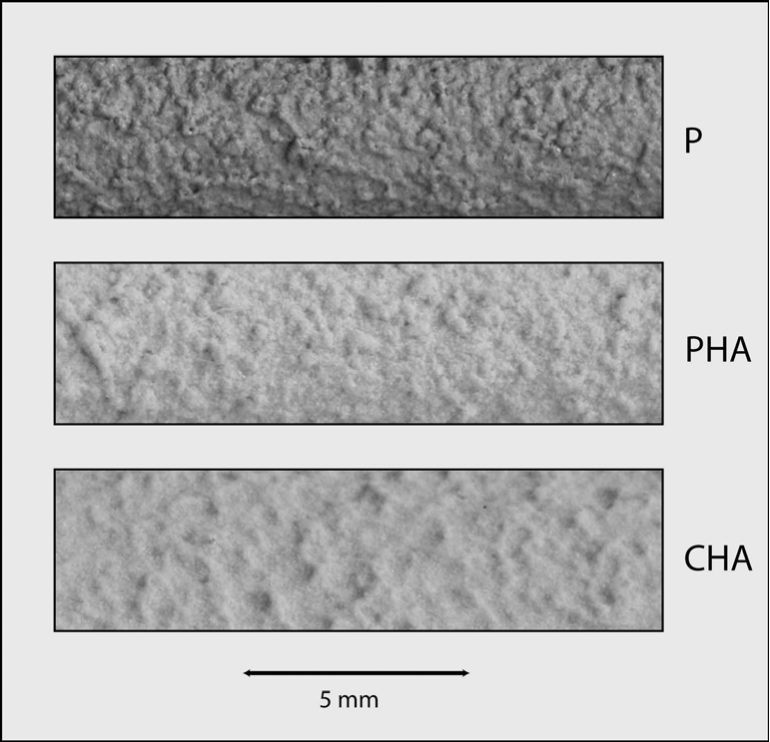
**Photographs of implant surfaces**. **P **= plasma-sprayed titanium (mean R_a _= 27 microns); **PHA **= plasma-sprayed titanium with plasma-sprayed hydroxyapatite coating (mean R_a _= 17 microns); **CHA **= chemical-textured titanium surface (by acid etching) with hydroxyapatite coating (mean R_a _= 26 microns). On visual inspection the surface texture of the P surface appear qualitatively more similar to the PHA surface when compared to the CHA surface.

Implants for intramedullary implantation in rabbit femora were manufactured and sterilized by Stryker Orthopaedics, Mahwah, NJ. Each implant consisted of a cylinder 5 mm in diameter and 25 mm in length (Figure 1). One of three surface treatments was applied to each implant: plasma-sprayed titanium (P), plasma-sprayed titanium with hydroxyapatite (PHA), or chemical-textured titanium with hydroxyapatite (CHA). The hydroxyapatite coating was applied by plasma spraying high purity hydroxyapatite powders with tightly controlled particle size using Sulzer Metco Plasma Spray System. HA powders were injected with Argon as the carrier gas to produce coating with thickness ranging from 40-70 microns (nominal 50 microns). The coating had a minimum total crystallinity of 65%. The minimum HA fraction in the crystalline phase was 90%. The average tensile and shear strength of the coating were ≥ 34 MPa and ≥17 MPa respectively. The chemical texturing was performed by repetitive masking (with an acid resistant mask) and chemical milling with nitric and hydrofluoric acid. The details regarding the chemical texturing process and the osseointegration of chemical-textured implants have been previously reported [22]. Implant surface roughness was measured with a Sheffield Profilometer (Sheffield, Fond du Lac, WI).

Thirty adult male New Zealand White rabbits were used in our study. After institutional review board approval, rabbits underwent bilateral femoral intramedullary implantation under general anesthesia. All animals received Buprenorphine 0.03 mg/Kg IM immediately postoperatively, and 0.01 mg/Kg IM every 12 hours for three days. After that any animal demonstrating pain or discomfort received Buprenorphine 0.01 mg/Kg IM. All animals were allowed unrestricted cage activity, and food and water ad libitum. Temperature was maintained at 24°C and humidity at 70%. All rabbits tolerated the anesthesia and surgical procedure uneventfully. Recovery was quick and rabbits were usually ambulating without noticeable limp by postoperative day 7. One rabbit developed intestinal obstruction after ingesting surgical dressing and was euthanized 6 days before schedule. The femora were harvested from this rabbit and included in the SEM analysis.

**Figure 2:**
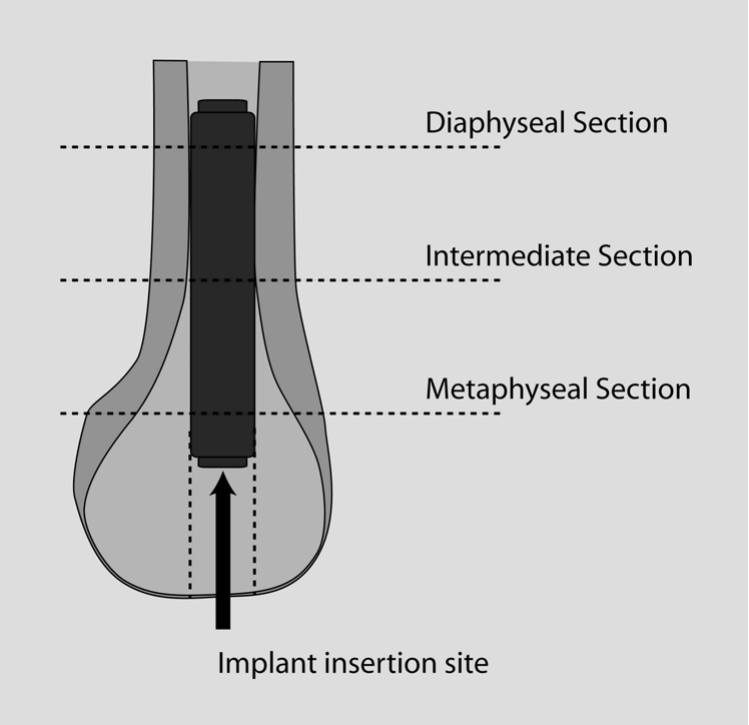
**Diagram of intramedullary implantation**. The implanted bone was sectioned at three levels shown.

The details of this in vivo rabbit model have been described previously (Figure 2) [22,28]. The appropriate experimental implant was press-fit into the intramedullary canal through a drill hole in the intercondylar notch of the femur. Bilateral implantation was used to reduce any bias introduced by unilateral implantation because the animal might favor the operated limb. Implants were distributed by type between limbs to permit paired comparison with an equal number of pairs per time point (P vs PHA, P vs CHA, and PHA vs CHA). Fifteen rabbits were euthanized postoperatively at 6 weeks; 15 at 12 weeks. At euthanasia, bilateral distal femora were harvested, cleaned of soft tissue, and fixed in 70% ethanol.

The femur bone was trimmed above and below the ends of the implant, cleaned of soft-tissue, and fixed in 70% alcohol. The specimen was further dehydrated in absolute alcohol and de-fatted in 50% mixture of ether and acetone before being placed in 100% alcohol again for 12 hours. The specimen was then embedded in methyl methacrylate and transverse sections nominally 1-mm thick cut with a diamond wafering blade at three levels, approximately coinciding with the distal third of the femoral diaphysis, the distal femoral metaphysis, and a level midway between the two. Backscatter electron images were obtained using a scanning electron microscope (JEOL 35, JEOL Ltd, Tokyo, Japan) at 40 × magnifications, 25-KeV beam voltage, and 100 μA emission current at a working distance of 15 mm. Images were of the implant-bone interface were captured around the perimeter of the implant and stored in 8-bit grayscale format at a resolution of 128 pixels per mm (pixel size 7.8 μm).

Automated computerized image analysis was performed on the SEM images using a previously validated approach [22,29]. A custom script was written (MATLAB, Image Processing Toolbox, MathWorks, Natick, MA). The image was segmented into bone and implant regions based on the trimodal histogram of the image. Images were initially filtered to remove random stray pixels. The image was segmented into three areas represented by: implant pixels (grayscale value between 200 and 255), bone pixels (grayscale value between 80 and 200), and soft-tissue pixels (grayscale value between 0 and 80). An edge detection algorithm was used to detect pixels at the perimeter of the implant and the bone and soft-tissue pixels adjacent to the edge of the implants were counted.

Osseointegration was defined as bone-to-implant contact and calculated as the ratio of the number of bone pixels relative to the total number of pixels (bone + soft tissue) at the perimeter of the implant. Additionally, the relative numbers of bone pixels were measured at varying distances (up to 0.24 mm) radially outward from the perimeter of the implant to detect changes in patterns of bone growth among the different surfaces.

Power analysis determined that a sample size of 10 was adequate to detect differences in osseointegration of greater than 15% among groups with a power greater than 80% and an alpha of 0.05, assuming a standard deviation of up to 11%. Results from four quadrants were averaged to obtain the net osseointegration and presence of bone for each section level.

Multifactorial two-way Analyses of Variance (ANOVA) were performed on mean osseointegration (or presence of bone at 0.03 to 0.24 mm from the implant surface) with surface treatment, time after surgery, and bone section level as the variables. When statistical differences were identified, Tukey post hoc pairwise comparisons were performed. Significant differences were assumed at p ≤ 0.05.

## Results

**Figure 3:**
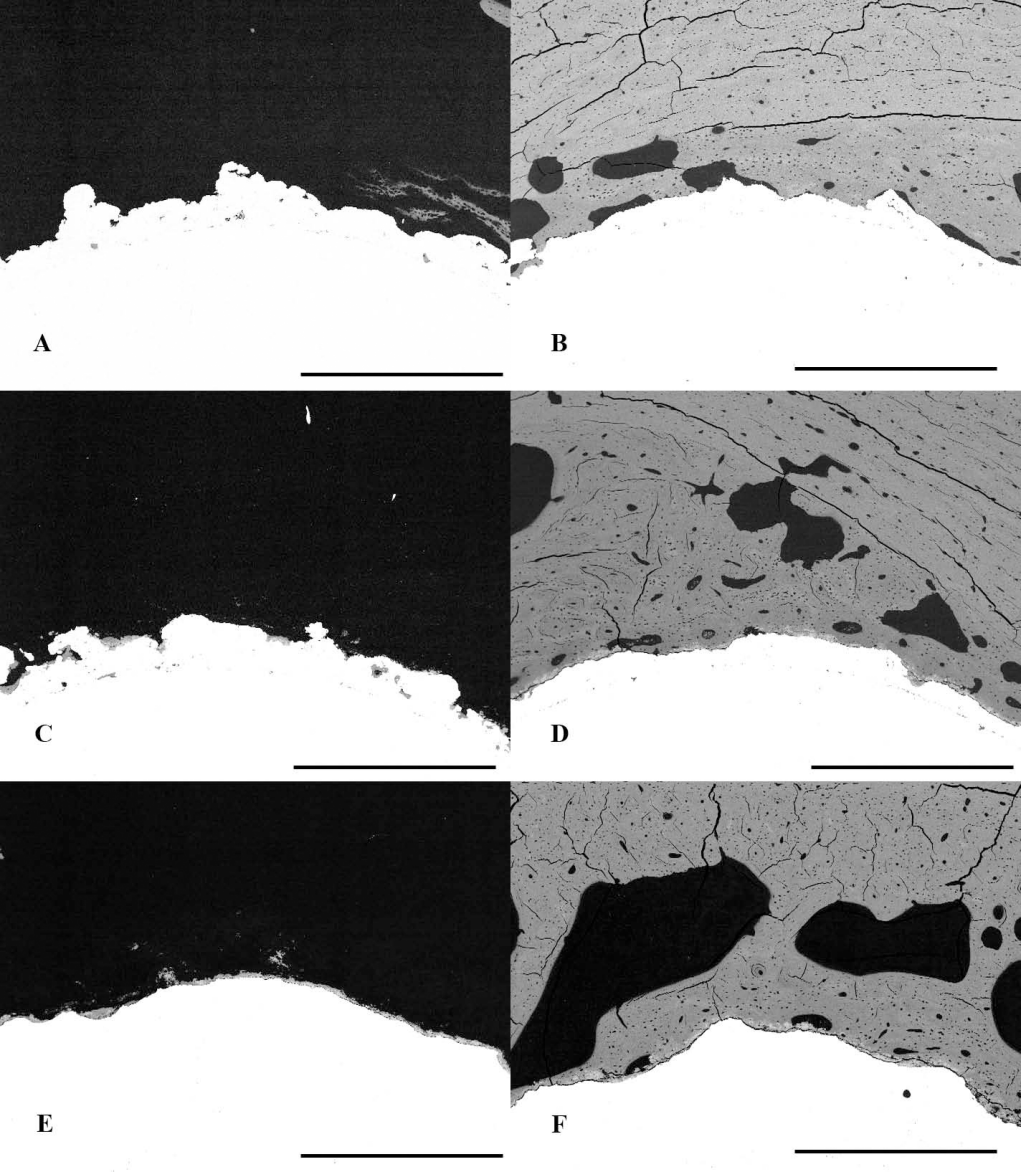
**Representative SEM images are shown depicting the range of low and high osseointegration for each surface**. **A**: Plasma-sprayed titanium surface (P) showing 0% osseointegration (intermediate level, posterior quadrant). **B**: Plasma-sprayed titanium surface (P) showing 46% osseointegration (diaphyseal level, anterior quadrant). **C**: Plasma-sprayed titanium surface with hydroxyapatite (PHA) coating showing 11% osseointegration (intermediate level, anterior quadrant). **D**: Plasma-sprayed titanium surface with hydroxyapatite (PHA) coating showing 100% osseointegration (diaphyseal level, anterior quadrant). **E**: Chemical-textured surface with hydroxyapatite coating (CHA) showing 24% osseointegration (intermediate level, anterior quadrant). **F**: Chemical-textured surface with hydroxyapatite coating (CHA) showing 97% osseointegration (diaphyseal level, anterior quadrant). The bar represents 1 mm (image resolution = 280 pixels per mm).

**Figure 4:**
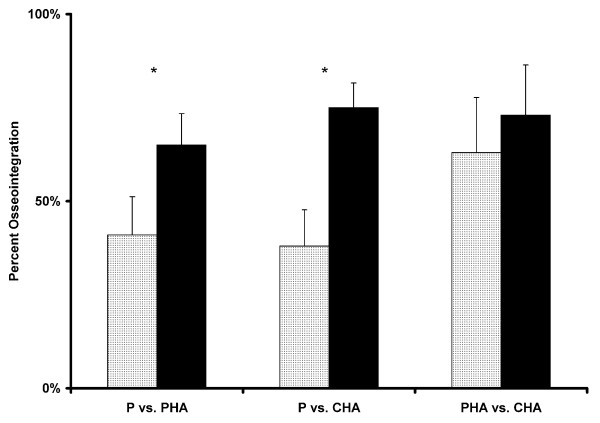
**Mean osseointegration (with standard deviation bars) was plotted for each paired comparison**. Data from 6 and 12 week time points were pooled. The hydroxyapatite-coated groups (PHA and CHA) consistently resulted in higher levels of osseointegration than in the uncoated group. The difference between the two hydroxyapatite-coated groups was not significant. (P = plasma-sprayed titanium; PHA = plasma-sprayed titanium with hydroxyapatite coating, and CHA = chemical-textured titanium with hydroxyapatite; * denotes statistically significant difference at p < 0.05)

**Figure 5:**
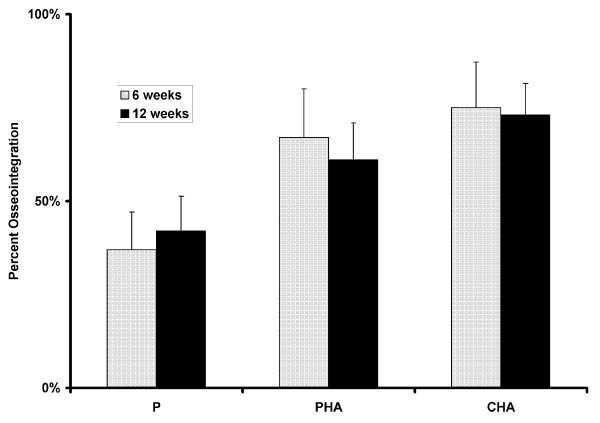
**Mean osseointegration (with standard deviation bars) was plotted for each group at the 6-week and 12-week time points**. No significant differences between time points were noted.

Mean surface roughness (R_a_) was 27 microns for the P group, 17 microns for the PHA group, and 26 microns for the CHA group (statistically different between the P and PHA groups and between the P and CHA groups). Representative SEM images of osseointegration for the three surfaces are shown in Figure 3. ANOVA indicated significant differences in osseointegration as a function of both section level and surface treatment. Mean osseointegration was significantly higher in the CHA (74 ± 15%) and PHA (64 ± 14%) groups as compared to the P group (39 ± 17%) (Figure 4). When all implant surfaces were pooled together, osseointegration at the diaphyseal level (69 ± 18%) was significantly greater than at both the intermediate (53 ± 22%) and metaphyseal levels (56 ± 19%). However, the differences in osseointegration along the axial direction were statistically similar between surface treatments (i.e., diaphyseal osseointegration was greater for all implant surfaces). No significant differences between 6 week and 12 week data were observed (Figure 5).

**Figure 6:**
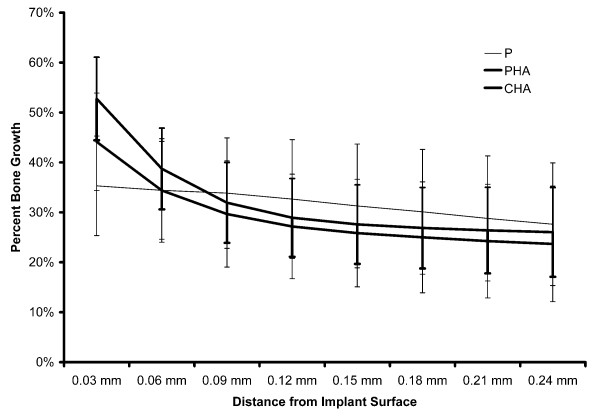
**Percentage of bone plotted as a function of distance from implant surface**. Six and 12 week data are pooled for each group. Bone growth was higher within 0.03 mm of the implant surface in the hydroxyapatite-coated groups compared to the uncoated group.

ANOVA also indicated significant differences in presence of bone radially outward from the perimeter of the implant. These differences were also related to both section level and surface treatment, with no time effect. Significantly greater bone was present within 0.03 mm of the implant surface was observed in the hydroxyapatite-coated groups (Figure 6). However, from 0.03 to 0.24 mm no further differences in presence of bone were noted as a function of surface treatment. Significant differences in presence of bone among bone section levels were also observed and these differences remained constant throughout the 0.24 mm distance from the implant perimeter evaluated. The presence of bone in the radial direction at the diaphyseal and metaphyseal levels was significantly higher than at the intermediate level. No significant differences in presence of bone were observed between 6 and 12 weeks.

## Discussion

The intramedullary bone response to three titanium surfaces (grit-blasted, porous fiber mesh, and acid-etched) was previously evaluated using the same animal model [22]. In that study, the chemically textured (by acid-etching) surface with a R_a _of 18 microns showed higher osseointegration than the grit-blasted surface with and R_a _of 6 microns. This study builds on our previous findings by investigating the effect of hydroxyapatite coating on surfaces with different roughness. The PHA and CHA groups had very different R_a _values of 17 microns and 26 microns, respectively, yet the osseointegration of each hydroxyapatite-coated surface was comparable, which suggested that the presence of the osteoinductive hydroxyapatite coating had a greater influence on bone growth than the surface roughness. Conversely, the mean R_a _values for the P and CHA groups were very similar at 27 microns and 26 microns, respectively. However, the osseointegration and distribution of bone were significantly different between these two groups.

Both surface roughness and hydroxyapatite coating have been shown to increase osseointegration [30]. Some reports have attributed increased osseointegration to surface roughness [23,31,32] while other reports to the hydroxyapatite coating [33-36]. Since the hydroxyapatite coating alters the surface roughness, a few studies have attempted to quantify the relative contribution of surface topography versus hydroxyapatite coating. Carlsson et al implanted titanium implants in the upper tibia of osteoarthritic knees of patients scheduled for total knee arthroplasty [37]. The osseointegration reported at 3 months was significantly higher in grit-blasted implants (mean R_a _= 3.1) than in implants with a smooth surface (mean R_a _= 0.9). This osseointegration was similar to that seen in implants coated with hydroxyapatite (mean R_a _= 5.1). However, the sample size studied was small with a large variance in the reported data. In a more controlled canine femoral intramedullary model, Hacking et al determined the relative contributions of surface chemistry and topography on osseointegration [26]. The hydroxyapatite surface of one group of implants was coated with a thin film of titanium, which masked the chemical activity of the hydroxyapatite coat while retaining the topography and surface roughness. Mean osseointegration of hydroxyapatite-coated implants (74%) was higher than the masked hydroxyapatite group (59%) or the grit-blasted group (23%). The relative increase in osseointegration between masked hydroxyapatite implants and grit-blasted implants was larger than the increase in osseointegration between hydroxyapatite-coated and masked hydroxyapatite implants. The authors therefore concluded that surface topography was the dominant factor influencing bone growth.

On the other hand, our study found a stronger correlation between the presence of hydroxyapatite and osseointegration than between surface roughness and osseointegration. In our study, the surface roughness of the implants used ranged from a R_a _of 17 to 26 microns. The surface roughness of the implants tested by Carlsson et al and Hacking et al were in the 3 to 6 micron range. It is therefore possible that an interaction effect exists between surface roughness and hydroxyapatite coating on osseointegration. At higher magnitudes of surface roughness, the hydroxyapatite coating may contribute more to osseointegration. The differences in findings underscore the need for additional research to better understand the processes that influence osseointegration.

Osseointegration was significantly higher at the diaphyseal level compared to that at the metaphyseal or intermediate levels. Implant-bone contact as well the type of bone (trabecular versus lamellar) varies along the axial direction. However, the differences in osseointegration along the axial direction were statistically similar between surface treatments. This suggests an absence of interaction effect between surface chemistry and location of implant. The presence of bone in the radial direction also varied by implant surface. Significantly greater bone was present within 0.03 mm of the implant surface in the hydroxyapatite-coated groups. While the SEM could not differentiate between newly deposited bone and pre-existing bone, these differences near the implant-bone surface were likely due to new bone formation.

The similarity in the chemistry of the hydroxyapatite coating with the crystalline phase of bone is believed to be one of the reasons for its excellent biocompatibility and osteoconductive properties. The slow but finite dissolution rate of crystalline hydroxyapatite provides a continuous source of calcium and inorganic phosphate [18]. In our present study, as well as in those reported by others, bone often appears to be directly deposited on the hydroxyapatite coating without any intervening layer of fibrous tissue, the latter being more commonly seen in uncoated titanium surfaces [22,23,28,37]. While hydroxyapatite by itself is considered osteoconductive, in vivo the surface adsorption of proteins (such as bone morphogenetic proteins) may render the surface osteoinductive [38,39]. In addition, osteoblasts may attach and release active osteoinductive factors[18]. All of these factors combined may be responsible for the enhanced bone response.

Clinical outcomes have substantiated the results of this animal model. Early osseointegration and more stable implant-bone interfaces were seen radiographically. In patients implanted with hydroxyapatite-coated femoral stems, no evidence of mechanical failures or progressive radiolucencies was noted [40,41]. Evidence exists that hydroxyapatite provides benefits beyond promoting osseointegration and enhancing implant stability. More complete osseointegration may act as a barrier to the migration of polyethylene debris along the bone-implant interface thereby reducing the incidence of osteolysis [9,10,42,43]. Rahbek et al demonstrated that hydroxyapatite effectively prevented particle migration when compared to non-coated grit-blasted titanium alloy implants in a canine femoral model [10,43,44]. A ten-year clinical follow up of a hydroxyapatite-coated femoral stem did not find evidence of distal osteolysis despite relatively high polyethylene wear [41,45]. With current-generation implant designs, short-term stability is no longer a major issue [14,15,46,47]. Longer-term follow up, however, shows polyethylene wear and lysis to be a major concern [48-51]. Measures that directly reduce wear (such as crosslinked polyethylenes and alternative bearing surfaces) have been introduced with some success [52,53]. However, a higher level of osseointegration is also extremely valuable, because it can reduce the incidence of distal osteolysis, which is one of the primary causes of implant failure [41,48,54].

One limitation of the study was the use of only roughness parameter (R_a_). Other roughness and surface parameters may also be important in determining potential for osseointegration. Osseointegration was only measured using one histomorphometric parameter (bone-to-implant contact). We did not measure the mechanical strength of the interface that is relevant for hip arthroplasty. However, others have correlated mechanical pull-out strength with the histomorphetric assessment of osseointegration [28].

Effective osseointegration of noncemented components plays an essential role in implant fixation, long-term stability, and survivorship. Our in vivo study evaluated the bone response to three surfaces, which adds to the body of evidence that is useful for optimizing the osseointegration of implants and enhancing fixation. It is important to identify factors that minimize joint arthroplasty failure and the significant physical and financial costs that failure represents. Finally, clinical outcomes studies are needed to validate the impact of implant surface and related osseointegration on THA outcomes.

## Competing interests

Research funds in support of this study were provided to Scripps Clinic from Stryker Orthopaedics. Two of the authors are employees of Stryker Orthopaedics.

## Authors' contributions

DDD, CWC, MH contributed to the conception and the study design. JCH, AB, MR participated in the data acquisition. DD and FD performed the data verification. FD, MH, CWC, DDD were involved in the data interpretation. JCH, MH, DDD contributed to the writing of the manuscript. All authors have read and approved the final manuscript.
